# Deep-Learning-Based Reduced-Order Model for Power Generation Capacity of Flapping Foils

**DOI:** 10.3390/biomimetics8020237

**Published:** 2023-06-05

**Authors:** Ahmad Saeed, Hamayun Farooq, Imran Akhtar, Muhammad Awais Tariq, Muhammad Saif Ullah Khalid

**Affiliations:** 1 Department of Mechanical Engineering, NUST College of Electrical & Mechanical Engineering, National University of Sciences & Technology, Islamabad 46000, Pakistan; 2 Department of Mathematics and Statistics, Institute of Southern Punjab (ISP), Multan 60800, Pakistan; 3 Department of Mechanical Engineering, Lakehead University, Thunder Bay, ON P7B 5E1, Canada

**Keywords:** power generation, long-short-term neural network, proper orthogonal decomposition, flapping foils, reduced-order modeling

## Abstract

Inspired by nature, oscillating foils offer viable options as alternate energy resources to harness energy from wind and water. Here, we propose a proper orthogonal decomposition (POD)-based reduced-order model (ROM) of power generation by flapping airfoils in conjunction with deep neural networks. Numerical simulations are performed for incompressible flow past a flapping NACA-0012 airfoil at a Reynolds number of 1100 using the Arbitrary Lagrangian–Eulerian approach. The snapshots of the pressure field around the flapping foil are then utilized to construct the pressure POD modes of each case, which serve as the reduced basis to span the solution space. The novelty of the current research relates to the identification, development, and employment of long-short-term neural network (LSTM) models to predict temporal coefficients of the pressure modes. These coefficients, in turn, are used to reconstruct hydrodynamic forces and moment, leading to computations of power. The proposed model takes the known temporal coefficients as inputs and predicts the future temporal coefficients followed by previously estimated temporal coefficients, very similar to traditional ROM. Through the new trained model, we can predict the temporal coefficients for a long time duration that can be far beyond the training time intervals more accurately. It may not be attained by traditional ROMs that lead to erroneous results. Consequently, the flow physics including the forces and moment exerted by fluids can be reconstructed accurately using POD modes as the basis set.

## 1. Introduction

Despite enormous advancements in computer-related technologies in the modern era, performing numerical simulations of complex flows using computational fluid dynamics (CFD) based tools demands a lot of resources in terms of computing time and data storage capacity. To cope up with these challenges, the idea of developing reduced-order models (ROMs), capturing the dynamics of engineering systems, is vital. The essence of such ROMs is to simulate the behavior of a system for a chosen set of values for the control parameters in the governing mathematical equations. For systems involved with fluid flows, the actual models usually consist of a system of nonlinear partial differential equations (PDEs). When the solutions are computed for a specific set of parameters, we can build *basis functions*. These functions help generate approximate solutions for new values of the governing parameters inexpensively. With the advantages offered by different classes of ROMs, such models were extensively developed for numerous applications, involving optimizations and control of complex engineering systems [1,2,3,4,5]. The success of a ROM highly depends on the choice of *basis functions* to be used to approximate the solution. Although a variety of dimensionality-reduction techniques exist, most ROMs are based on proper orthogonal decomposition (POD) technique [6,7] serving as the *basis functions*. The POD method is very effective for dimensionality reduction complex models of physical systems. Snapshots (measurements) of many nonlinear dynamical systems often exhibit low-dimensional activity, and their contribution in a whole phenomenon is minimal. It is because most of the energy (quantified through the variance of a signal) is contained in a few modes computed through singular value decomposition (SVD) or method of snapshots [8]. Such modes serve as the *reduced-order basis* to construct the low-dimensional space for the construction of a ROM. The significance of the POD method in the development of ROMs can be found in numerous fields, including complex flows [9], flow control design [1,10,11,12,13], pattern recognition [8], and uncertainty quantification and optimization [14]. The application of such POD-ROMs in turbulent flows is not straightforward. Some recent pieces of work on closure modeling attempts to extend the application of POD-ROMs in complex flows [9,15,16,17,18]. Generally, the construction of a POD-ROM is a two-step process: (a) post-processing of flow field data to compute the optimal basis functions, and (b) Galerkin projection of the governing equations onto these basis functions to develop a ROM. Linear combinations of POD modes and expansion coefficients (temporal coefficients) are employed to represent a time-evolving flow field. POD-Galerkin-based models suffer from issues related to instability and efficiency [18,19,20,21,22,23].

Although a significant reduction in computational time and cost may be attained to find the solution of complex phenomena by using ROMs, there exists a lot of room for researchers to formulate better alternates for further improvements. The inclusion of machine learning (ML) in CFD brings a revolution in this field [24,25,26,27]. Han et al. [28] introduced a deep-learning-based approach for solving highly complex systems of PDEs. They used neural networks to predict the values of unknown parameters and concluded that the solver performed well in terms of accuracy and computational cost. Hesthaven and Ubbiali [29] utilized ANN to approximate the coefficients of a reduced-order model for parameterized steady-state PDEs. Their results confirmed the speedup and accuracy of the ANN-based model.

Most ROMs based on the POD technique employ information about velocity fields [5,21] whereas, the pressure field, specifically on the surface, also plays an important role in the accuracy of POD-based ROMs [30]. Many studies were carried out for ROMs developed based on pressure mode decomposition (PMD) method that also enabled accurate estimations of hydrodynamic forces. Recently, Ahmed et al. [31] used PMD technique to develop an ML-based ROM to predict the lift and drag forces for flows around circular cylinders. Later, Farooq et al. [32] developed an ANN-based ROM to approximate the hydrodynamic forces on a NACA-0012 airfoil exerted by the fluid flows, passing over it at different angles-of-attack (AoA). The trained model produced accurate values of lift and drag forces that match well with the true values obtained through direct numerical simulations. Despite vast research in this field, the current situation demands more novel ideas and techniques stemming from ANN-based ROMs for accurate prediction of hydrodynamic forces on flapping foils similar to the conventional ROMs, where the solution can be predicted for a long time duration.

Most of the research studies of flapping foils/wings [33,34,35,36,37] are focused more to examining biological and bio-inspired propulsion mechanisms with the primary objective of developing efficient engineered propulsive devices. Alternatively, the flapping foils/wings can also be employed to harness energy from the fluid flows, passing over them. Flapping foils-based power extraction systems are usually classified into three categories according to their activation mode [38,39]: (i) a fully forced system in which both plunge and pitch motions are prescribed [38,40,41,42,43,44]; (ii) a semi-passive system in which the pitch motion is prescribed while the plunge motion is induced from the interactions between the foil, the incoming flow, and the elastic supports [45,46,47,48,49,50]; and (iii) a fully-passive system in which both plunge and pitch motions are entirely driven by interactions of the foil with the surrounding fluid and the supporting dynamical mechanisms [42,44,51,52,53,54]. In this work, we consider a fully forced system for developing a deep-learning-based ROM for power generation by flapping foils. Generally, the power extraction performance of a flapping foil is measured through a parameter called power extraction efficiency [41]. Kinsey and Dumas [41] conducted a numerical parametric study to investigate the performance of the fully forced system of a NACA-0015 airfoil at a Reynolds number (Re) of 1100 for the range of flapping frequency and pitching amplitude. The main objective of their study was to determine the ranges for the optimal frequency and pitching amplitude for maximum power extraction efficiency. Their study demonstrated that the power extraction efficiency could exceed 20% when setting the pitching amplitude greater than $55^{\circ }$. It reaches its maximum value of 34% when considering the plunging amplitude equal to the foil’s chord length and selecting the pitching amplitude higher than $75^{\circ }$. In another numerical study conducted by Ashraf [55], the power extraction efficiency of NACA-0014 airfoil at Re = 20,000 was examined. He investigated the effect of the phase angle between the plunge and pitch kinematics. The peak value of the power coefficient and efficiency (32%) was achieved at the phase angle of $95^{\circ }$ with a plunging amplitude equal to 5% more than the foil’s chord length. Recently, Farooq et al. [40] conducted a numerical study to investigate the power extraction performance of a fully forced NACA-0012 airfoil at Re=1100. They performed a parametric study by varying the Strouhal number and the amplitude of the pitching angle to identify two operational flow regimes: power generation and thrust-producing propulsion using the feathering criterion [41]. Their parametric study revealed that the foil could reach up to 42% power generation efficiency when setting the pitching amplitude in the range of $60^{\circ }$ to $70^{\circ }$ with Strouhal number synchronized with the non-dimensional excitation frequency. Moreover, they identified the locations where the fluid pressure was dominant during the oscillating cycle of the flapping airfoil, operating in the power generation regime and designed a piezoelectric energy harvester for electrical power production [56].

The novelty of our present research is to develop and employ a recurrent neural network (RNN) [57] model to predict temporal coefficients of the pressure modes. These coefficients, in turn, are used to reconstruct hydrodynamic forces and moment, leading to computations of power. It is important to mention here that, in a conventional ROM, temporal coefficients are evaluated by integrating the time-evolving dynamical system, which is usually a set of initial-valued ordinary differential equations and can lead to erroneous results for complex flows. However, in ML-based ROMs, these temporal coefficients can be predicted accurately by employing an RNN-based model [58,59]. Note that the pressure temporal coefficients are not only required for the reconstruction of hydrodynamic forces and moment but also are useful for the development of efficient ROMs for shear flows [30]. The main focus of the current study is to develop a ROM for a power generation system using a variant long-short-term neural network (LSTM) for the prediction of temporal data. Our newly proposed model is very similar to a traditional ROM, which takes the known temporal coefficients as input and predicts the future temporal coefficients followed by previously estimated temporal coefficients. Consequently, we can predict the temporal coefficients for a long time duration that can be far beyond the training time intervals more accurately, which may not be attained by traditional ROMs that lead to erroneous results. The model’s efficiency is verified by reconstructing hydrodynamic forces and moment accurately. Note that for reconstruction, we do not employ the training temporal data. The motivation behind the choice of the LSTM model compared to others is that LSTM models are particularly well-suited for tasks that require modeling sequential data with long-term dependencies. These models were previously shown to perform well in a wide range of applications for reduced-order modeling of fluid flow problems [60,61,62,63], which made them a popular choice in many deep-learning tasks. LSTM models have some advantages over other models, such as for capturing long-term dependencies. These techniques can overcome the “vanishing gradient" problem, as explained in Section 5.3. Handling variable input sequence length, they do not need fixed inputs and output sizes, unlike *feedforward* neural networks. Having memory and recurrent connections, they models can store and propagate information over time steps, which is useful for tasks, requiring temporal dependencies.

The remaining manuscript is organized as follows. The governing mathematical models for incompressible two-dimensional flows over flapping foils are presented in Section 2. In addition, important details about our computational techniques are presented here. Next, the validation studies of our numerical methodology is provided in the Section 4. Details on the feature extraction technique, POD of the pressure fields, using the method of snapshots and the ANN training model and its behavior constitute Section 5.

## 2. Numerical Methodology

### 2.1. Governing Formulations for Incompressible Fluid Flows

The mathematical model, describing the two-dimensional (2D) unsteady incompressible flows around flapping airfoils consists of the continuity and Navier-Stokes equations, which are defined in their respective tensor as given below:

(1) $$\frac{\partial u_{i}}{\partial x_{i}}=0$$

(2) $$\frac{\partial u_{i}}{\partial t}+u_{j}\frac{\partial u_{i}}{\partial x_{j}}=-\frac{1}{\rho }\frac{\partial p}{\partial x_{j}}+\frac{1}{Re}\left(\frac{\partial^{2}u_{i}}{\partial x_{j}^{2}}\right)$$

where the $u_{i}$ represents the fluid velocity, *p* is the pressure, and ρ is the density of the fluid. This governing model (Equations (1) and (2)) is provided in the nondimensional form, where nondimensionalization is carried out by considering the free-stream uniform velocity ($U_{\infty }$) as the velocity scale and the chord-length (*c*) of the airfoil as the length scale. Thus, the Reynolds number (Re) is defined as $Re=\rho cU_{\infty }/\mu$, where μ is dynamic viscosity of the fluid.

In order to handle the effects of moving bodies in a flowing fluid, we employ *Arbitrary Lagrangian–Eulerian* (ALE) approach in which the momentum equation (Equation (2)) is modified as:

(3) $$\frac{\partial u_{i}}{\partial t}+{\tilde{u}}_{j}\frac{\partial u_{i}}{\partial x_{j}}=-\frac{1}{\rho }\frac{\partial p}{\partial x_{j}}+\frac{1}{Re}\left(\frac{\partial^{2}u_{i}}{\partial x_{j}^{2}}\right)$$

**Figure 1:**
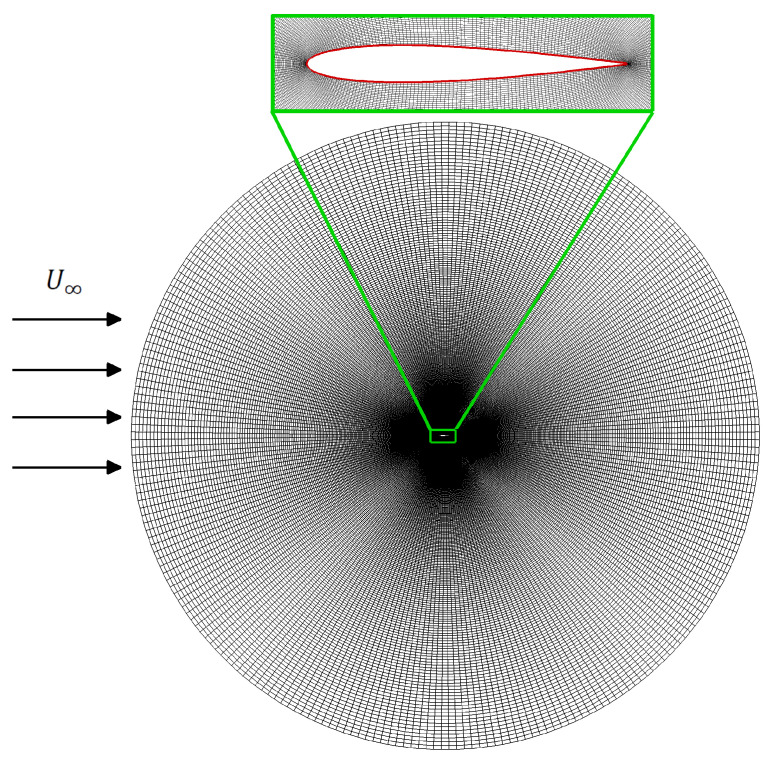
Two-dimensional layout of an ‘O’-type body-fitted grid over the NACA0012 airfoil. Here, the horizontal arrows show the direction of the incoming flow.

Here, the term ${\tilde{u}}_{j}$ represents the relative velocity of the fluid with respect to the corresponding grid node-velocity, that is, ${\tilde{u}}_{j}=u_{j}-u_{g_{j}}$. The term $u_{g_{j}}$ represents the grid node velocity, which has to be computed at each time level. Note that Equations (2) and (3) will becomes identical for $u_{g_{j}}=0$, which shows *Eulerian* description and the underlying grid thus remains fixed. On the other hand, for $u_{j}-u_{g_{j}}=0$, Equation (3) then represents the *Lagrangian* description of flow dynamics. Typically, the success of the ALE approach depends on the strategy for how the computational grid deforms during simulations. In this study, we employ the *radial basis function* (RBF) interpolation technique, for grid deformations. This method was originally proposed and developed by [64], and it is known for its robustness, accuracy, and its capability to maintain good grid quality even for large structural translations and rotations [65]. Moreover, the computation grid used here is an ‘O’-type body-fitted grid around a NACA-0012 airfoil, as shown in Figure 1. Note that we consider a grid of size 400×304 with 400 points in the radial direction and 304 points on the surface of the airfoil. The outer radius of the circular domain around the airfoil is 30c. Moreover, the same grid size and resolution are used to compute the POD modes.

Because the computational domain has curved grid lines, the following expressions are used to transform the governing equations into the curvilinear coordinates (α,β), depending on the Cartesian coordinates (x,y):

(4) $$\alpha =\alpha \left(x,y\right),\;\;\;\;\;\;\;\;\beta =\beta \left(x,y\right)$$

Thus, after some mathematical manipulations and simplifications, we obtain the following form:

(5) $$\begin{array}{cc} \frac{\partial U_{m}}{\partial \alpha_{m}} & =0, \end{array}$$

(6) $$\begin{array}{cc} \frac{\partial (G^{-1}u_{i})}{\partial t}+\frac{\partial F_{im}}{\partial \alpha_{m}} & =0, \end{array}$$

where the flux is defined as

(7) $$F_{im}={\tilde{U}}_{m}u_{i}+G^{-1}\frac{\partial \alpha_{m}}{\partial x_{i}}p-\frac{1}{Re}Q^{mn}\frac{\partial u_{i}}{\partial \alpha_{n}}.$$

where $G^{-1}=det\left(\frac{\partial x_{i}}{\partial \xi_{j}}\right)$ is the inverse of the Jacobian or the volume of the cell; ${\tilde{U}}_{m}=G^{-1}\frac{\partial \alpha_{m}}{\partial x_{j}}{\tilde{u}}_{j}$ is the volume flux (contravariant velocity multiplied by $G^{-1}$) normal to the surface of constant $\xi_{m}$; and $Q^{mn}=G^{-1}\frac{\partial \alpha_{m}}{\partial x_{j}}\frac{\partial \alpha_{n}}{\partial x_{j}}$ is the “mesh skewness tensor”.

### 2.2. Prescribed Flapping Kinematics

The mathematical models for combined plunging and pitching (or flapping) kinematics are defined as:

(8a) $$\begin{array}{c} y\left(t\right)=A_{y}\sin\left(2\pi f_{e}t\right),\; \end{array}$$

(8b) $$\begin{array}{c} \dot{y}\left(t\right)=A_{y}\left(2\pi f_{e}\right)\cos\left(2\pi f_{e}t\right) \end{array}$$

(8c) $$\begin{array}{c} \alpha \left(t\right)=A_{\alpha }\sin\left(2\pi f_{e}t+\frac{\pi }{2}\right), \end{array}$$

(8d) $$\begin{array}{c} \;\dot{\alpha }\left(t\right)=A_{\alpha }\left(2\pi f_{e}\right)\cos\left(2\pi f_{e}t+\frac{\pi }{2}\right) \end{array}$$

where $A_{y}$ and $A_{\alpha }$ are the nondimensional plunging and pitching amplitudes, respectively and $f_{e}$ is the oscillating excitation frequency. The terms y(t) and α(t) represent the instantaneous plunging and pitching displacements of the airfoil. For these kinematic settings, the Strouhal number $\left({St}_{A}\right)$ here is defined as the ratio between the product of the oscillating frequency $\left(f_{e}\right)$ and peak-to-peak amplitude $\left(2A_{y}\right)$ of the trailing edge of the airfoil and the uniform velocity $\left(U_{\infty }\right)$, i.e., ${St}_{A}$=$2f_{e}A_{y}/U_{\infty }$. In our present study, we keep the value of $f_{e}$ fixed at 0.375Hz. Additionally, it is important to mention, the superscript (·) in Equation (8b),(8d) represents the time derivative.

### 2.3. Discretization Strategy

The fractional step method is an effective and strong candidate technique for dealing with incompressible Navier-Stokes equation. It transforms the momentum equation into the convection-diffusion equation and pressure-Poisson equation [32,40,51,66]. We utilize a non-staggered grid in which the velocity and pressure fields are computed at the center of each cell. The fluxes $\left(\tilde{U},\;\tilde{V}\right)$ are calculated on the corresponding faces at their midpoints. Except for the convective term, all the spatial derivatives are approximated using the second-order central difference method. The use of a central difference scheme for convective terms may result in unnecessary fluctuation and thus, may lead to incorrect or erroneous solutions. However, such issues can be resolved by implementing an appropriate higher-order difference scheme. For this purpose, an upwind scheme with quadratic upwinding interpolation for convective kinematics (QUICK) [67] is employed here to discretize the convective terms. To advance the solution in time, a hybrid method composed of explicit and semi-implicit schemes is used. The Crank–Nicolson (C-N) scheme is employed to discretize the diagonal viscous terms only, whereas all the remaining terms are discretized using the second-order Adams–Bash (AB-2) scheme. It is important to mention here that both these schemes are second-order accurate.

## 3. Hydrodynamic Performance Metrics

The hydrodynamic forces and moments on an oscillating body are produced due to pressure distribution and shear stress distribution over the surface of the body. The net effect of these terms integrated over the complete body surface gives us the resultant hydrodynamic force (*R*) and moment on the body, where *R* can be split into components; normal and axial forces, as illustrated below.

(9a) $$F_{N}=\oint_{c}dN=-\oint_{c}\left(p\;\sin(\theta )-\tau \;\cos(\theta )\right)\;ds,$$

(9b) $$F_{A}=\oint_{c}dA=-\oint_{c}\left(p\;\cos(\theta )+\tau \;\sin(\theta )\right)\;ds,$$

(9c) $$M=-\oint_{c}\left(x^{b}-x_{p}\right)dF_{N}+\oint_{c}\left(y^{b}-y_{p}\right)dF_{A},$$

where $F_{N}$ and $F_{A}$ are the axial and normal force components that are parallel and perpendicular to the chord of the airfoil, respectively. The term τ represents the shear stress, whereas *p* is the pressure. Furthermore, *M* is the pitching moment about the pitching axis point $\left(x_{p},\;y_{p}\right)$, and *s* is the arc-length of the body. Thus, the hydrodynamic lift (*L*) and drag (*D*) forces over the airfoil at the pitching angle α can be computed as:

(10a) $$L=F_{N}\cos\left(\alpha \right)-F_{A}\sin\left(\alpha \right),$$

(10b) $$D=F_{N}\sin\left(\alpha \right)+F_{N}\cos\left(\alpha \right)$$

The dimensionless forces and moment coefficients can be defined by using dynamic pressure ($q_{\infty }=\frac{1}{2}\rho U_{\infty }^{2}$) as follows:

(11a) $$\begin{array}{c} C_{L}=\frac{L}{q_{\infty }c}, \end{array}$$

(11b) $$\begin{array}{c} \;C_{D}=\frac{D}{q_{\infty }c}, \end{array}$$

(11c) $$\begin{array}{c} \;C_{M}=\frac{M}{q_{\infty }c^{2}}. \end{array}$$

## 4. Validation

**Figure 2:**
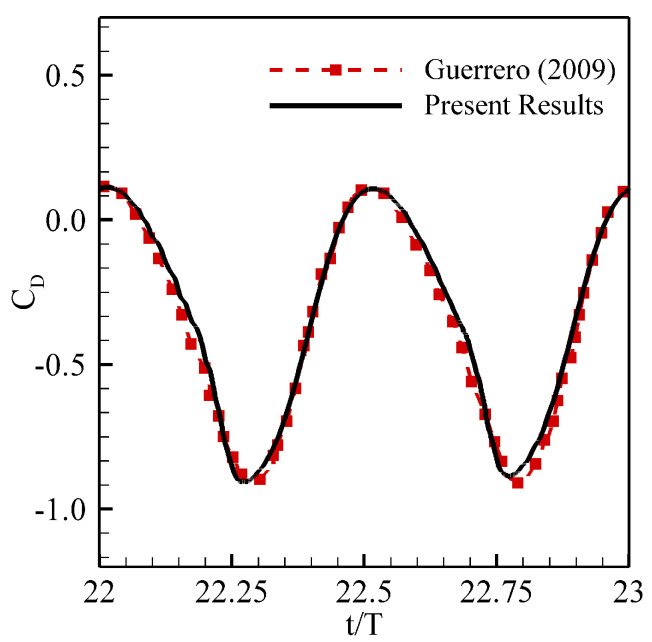
Validation of solver: Comparison of $C_{D}$ of the flapping airfoil with the temporal data of [37]. Here *T* represents the oscillation time period.

In order to demonstrate the effectiveness and accuracy of our in-house computational solver, we simulate the flows over the NACA-0015 airfoil, undergoing simultaneous plunging and pitching motions at Re=1100. We compute the hydrodynamic lift and drag coefficients and compare the temporal profile of $C_{D}$ with those reported by [37]. Here, kinematic amplitudes are $A_{h}=0.4$, $A_{\alpha }=20^{\circ }$, for plunging and pitching motions, respectively. Figure 2 presents that the results from our computational solver are in excellent agreement with those from [37]. A body-fitted nearly orthogonal grid of size 353×501 around the foil is used for our simulations. The outer boundary of this grid is located at a radius of 25c that is far from the surface of the foil, as shown in Figure 1). For simulating the flow over the moving body here, we use the time-step equal to $2\times 10^{-4}$. For more details on grid convergence and time-step independence, interested readers are referred to the Refs. [40,51].

## 5. Results and Discussions

In this study, we perform numerical simulations for incompressible flows over a NACA-0012 foil that is subjected to sinusoidal plunging and pitching (flapping) motions simultaneously at Re=1100. Before analyzing the accuracy of a deep-learning neural network-based ROM for the pressure field, we first discuss the performance of a flapping foil, operating in the power generation regime. Under these conditions, the directions of the lift force and plunging velocity are mostly the same in an oscillation cycle. Thus, the flapping foil experiences a positive work by the fluid, passing over it. Such positive work can be utilized for power extraction [40,41]. We simulate several cases from the parametric space of $\left({St}_{A},\;A_{\alpha }\right)=\left\{0.1\leq {St}_{A}\leq 0.35\;\mathrm{and}\;15^{\circ }\leq A_{\alpha }\leq 100^{\circ }\right\}$ and compute the power generation efficiency (η) defined as:

(12a) $$\begin{array}{c} \eta ={\bar{C}}_{P}\frac{c}{Y_{P}} \end{array}$$

(12b) $${\bar{C}}_{P}=\frac{1}{T}\int_{t}^{t+T}\left[C_{L}\frac{\dot{y}\left(t\right)}{U_{\infty }}+C_{M}\frac{\dot{\alpha }\left(t\right)c}{U_{\infty }}\right]\;dt$$

where $Y_{P}$ is the difference between the highest and the lowest points reached by the foil, and $T=1/f_{e}$ is the time period. Furthermore, the term $C_{P}$ represents the power coefficient that can be decomposed as $C_{P}=C_{P}^{p}+C_{P}^{\nu }$. Here $C_{P}^{p}$ denotes the power coefficient based on pressure only, whereas $C_{P}^{\nu }$ is the shear stress-based power coefficient.

**Figure 3:**
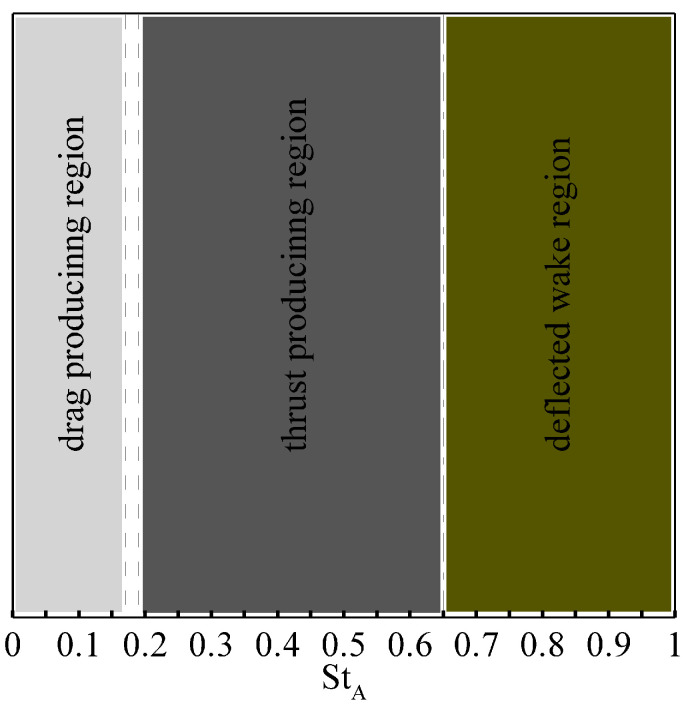
Operational regimes of flapping foil at a pitching amplitude of $A_{\alpha }=20^{\circ }$.

**Figure 4:**
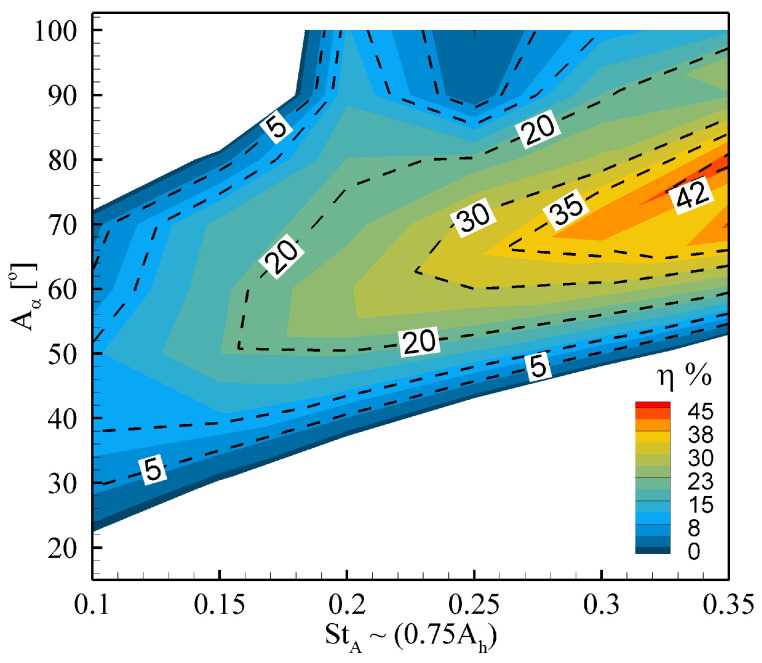
Contours of power generation efficiency in parametric space $\left({St}_{A},\;A_{\alpha }\right)$.

For the foil pitching with an amplitude of $A_{\alpha }=20^{\circ }$, we identify well-known operational regimes in the range of $0<{St}_{A}\leq 1.0$, including drag-production, thrust-production, and deflected wake regions (see Figure 3). Moreover, we observe that the neutral wake (i.e., the case for which the mean drag coefficient is zero) is formed at ${St}_{A}=0.18$. As the power generation efficiency of the foil is a concern, we show the variation in η in terms of a contour plot for the pitching amplitude $A_{\alpha }$ and Strouhal number ${St}_{A}$ in Figure 4. In this parametric space, we obtain a remarkable power efficiency of 42%. For comprehensive details about the hydrodynamic performance and the wake topology of the flapping foil, the readers are referred to read the following article [40].

**Figure 5:**
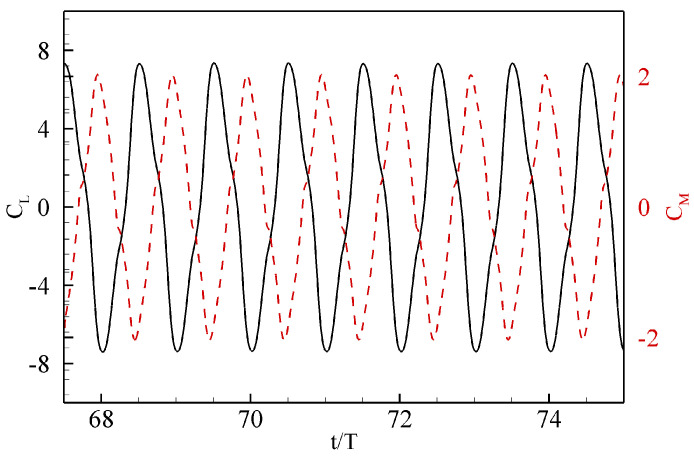
Time histories of lift (solid line) and pitching moment (dashed line) coefficients at ${St}_{A}=0.33$.

**Figure 6:**
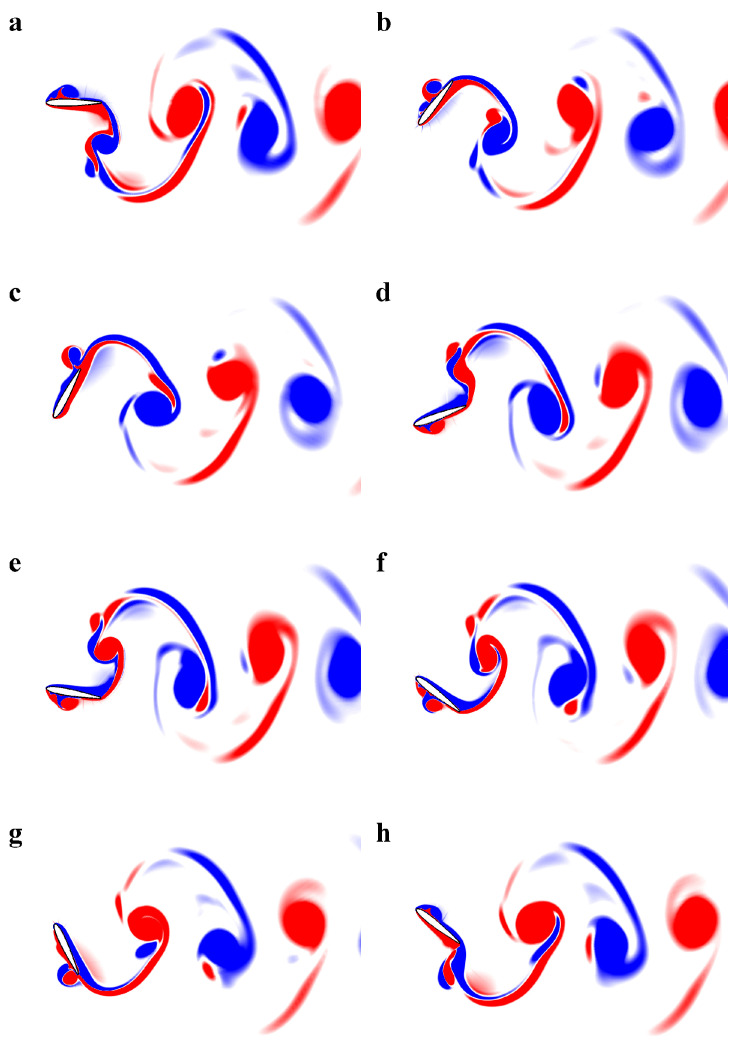
Spanwise vorticity contours over a complete oscillating cycle of flapping foil at ${St}_{A}=0.33$. Plots from one-half oscillating cycle are depicted in (**a**–**d**), which are corresponding to the time-instants from t/T=0 to 3/8 while the plots from the other half cycle are depicted in (**e**–**h**). Here, the symbol *T* represents the time period of an oscillating cycle.

Next, we develop a ROM for the pressure profiles over the flapping foil, exhibiting power generation mode [41] through pressure-based POD and deep-learning neural network techniques. Our primary objective is to reconstruct the forces and moments exerted by the fluid on the body through the trained deep-learning model more efficiently. For this purpose, we choose a case of a foil flapping with $A_{\alpha }$ as $67^{\circ }$ and ${St}_{A}=0.33$ (or $A_{h}=0.44$), because these kinematic parameters correspond to a remarkable power generation efficiency of 40% at Re=1100 (see Figure 4). We simulate this case for a long enough time duration in order to mitigate the transient effects and obtain the periodic steady state. The time histories of the lift and the pitching moment coefficients are shown in Figure 5. We observe a phase of $90^{\circ }$ between $C_{L}$ and $C_{M}$. We present the snapshots of vorticity contours over a complete oscillation cycle in Figure 6. From these plots, we observe a pair of vortices, formed at the leading-edge during the first half-cycle, and similar coherent flow structures produced during the other half-cycle. These pairs then interacts with those formed at the trailing-edge, resulting in a dominant single vortex in the wake. As reported earlier, the leading edge vortex dominates the output of positive power generation [41,47], we notice a similar phenomenon here. Next, we discuss the strategies for computing pressure POD modes and training a deep-learning neural network model to develop ROMs for hydrodynamic forces and moments in the following passages.

### 5.1. Proper Orthogonal Decomposition

Here, we elaborate the methodology to compute dominant coherent structures of the flow field. One of the commonly known techniques is the POD method to extract dominant modes of the pressure field. Generally, the POD modes can be computed through the procedure of either SVD or the method of snapshots [68,69]. In this study, we utilize the method of snapshots because it is computationally inexpensive compared to SVD for high-fidelity simulations. The set of ensemble *S* time-discrete snapshots $\left(p_{n}=p\left(x,t_{n}\right),t_{n}=t_{s}+\left(n-1\right)\Delta t,n=1,2,\cdot \cdot \cdot ,S\right)$ is used as an input for POD of a flow property, such as pressure. Moreover, the time-dependent flow field *p* can be decomposed into its mean $\bar{p}\left(x\right)$ and fluctuating part $p^{\prime }\left(x,t\right)$, and the fluctuating part $p^{\prime }\left(x,t\right)$ is expanded in a Galerkin fashion in terms of temporal and spatial variables, as given below:

(13) $$\begin{array}{c} p\left(x,t\right)=\bar{p}\left(x\right)+p^{\prime }\left(x,t\right)=\bar{p}\left(x\right)+\sum_{j=1}^{\infty }a_{j}\left(t\right)\varphi_{j}\left(x,t\right)\\ \approx \bar{p}\left(x\right)+\sum_{j=1}^{m}a_{j}\left(t\right)\varphi_{j}\left(x\right) \end{array}$$

where *m* represents the finite number of modes in the expansion, φ(x) represents the POD modes of *p* to be determined through the eigenvalue problem, and $a_{j}\left(t\right)$ denotes the temporal coefficients. Note that, in this study, our primary aim is to propose and train a deep-learning model to estimate $a_{j}$. The instantaneous solutions at the time $t_{1},t_{2},\cdot \cdot ,t_{s}\in \left(0,t\right)$ of *P* are stored in a matrix of size N×S, where S≪N, and *N* denotes the number of grid points. The problem is to seek a low dimensional basis $\left\{\varphi_{1},\varphi_{2}\cdot \cdot \varphi_{m}\right\}$ that satisfies the following relation:

(14) $$\begin{array}{c} min\frac{1}{S}\sum_{i=1}^{s}{∥p^{\prime }\left(\cdot ,t_{i}\right)-\sum_{j=1}^{m}{\left(p^{\prime }\left(\cdot ,t_{i}\right),\varphi_{j}\left(\cdot \right)\right)}_{H}∥}_{H}^{2} \end{array}$$

subject to the condition of orthogonality, ${\left(\varphi_{i},\varphi_{j}\right)}_{H}=\delta_{i,j},1\leq i,j\leq m,$ where $\delta_{i,j}$ is the Kronecker delta function and *H* is a real Hilbert space such that p(·,t)∈H|t∈(0,T). To solve Equation (14), we consider the following eigenvalue problem

(15) $$\begin{array}{c} Cv=\lambda v \end{array}$$

where $v_{k}$ for k=1,2,···,S are the eigenvectors, $\lambda_{1}\geq \lambda_{2}\cdot \cdot \cdot \geq \lambda_{S}>0$ are eigenvalues, and $C\in R^{S\times S}$ is the correlation matrix that can be defined as

(16) $$\begin{array}{c} C_{ij}=\frac{1}{S}{\left(p^{\prime }\left(\cdot ,t_{j}\right),p^{\prime }\left(\cdot ,t_{i}\right)\right)}_{H} \end{array}$$

It can be shown that the solution of Equation (14) is given by [68]

(17) $$\begin{array}{c} \varphi_{k}\left(\cdot \right)=\frac{1}{\sqrt{\lambda_{k}}}\sum_{j=1}^{S}{\left(v_{k}\right)}_{j}p^{\prime }\left(\cdot ,t_{j}\right),1\leq k\leq m \end{array}$$

**Figure 7:**
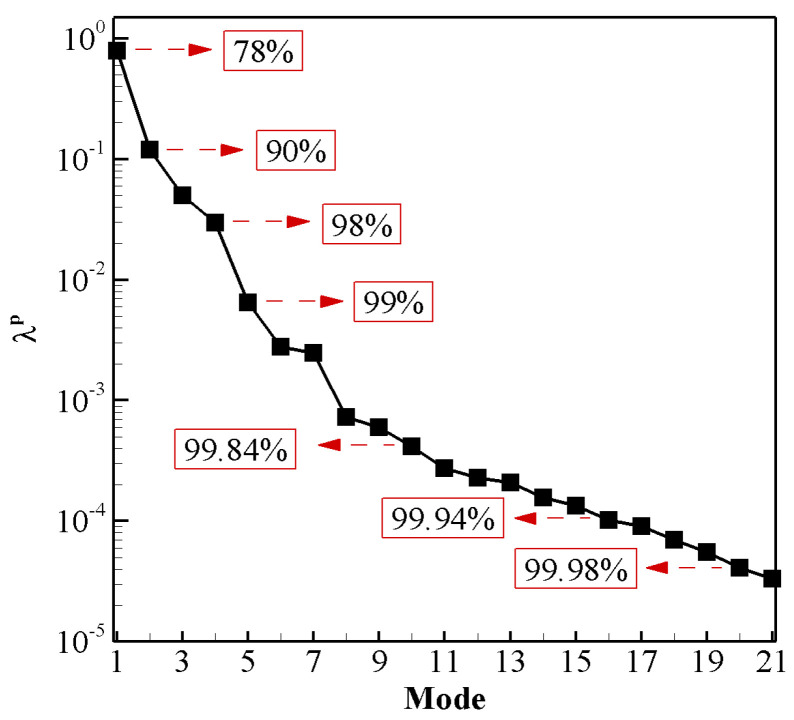
Normalized eigenvalues of the correlation matrix *C* and their corresponding contributions for the cumulative energy content.

We compute the normalized eigenvalues ($\lambda_{k}/\sum_{j}\lambda_{j}$) to exhibit the quantification of variance/energy in the POD modes. In Figure 7, the pressure-based eigenvalues ($\lambda^{p}$) are shown along with the cumulative amount of energy. It can be inferred that the first 20 POD modes contain almost 99.98% of total energy. Consequently, the first 20 modes should be enough for a low-dimensional model of the pressure field, and hence, of the hydrodynamic forces and moment based on pressure only.

**Figure 8:**
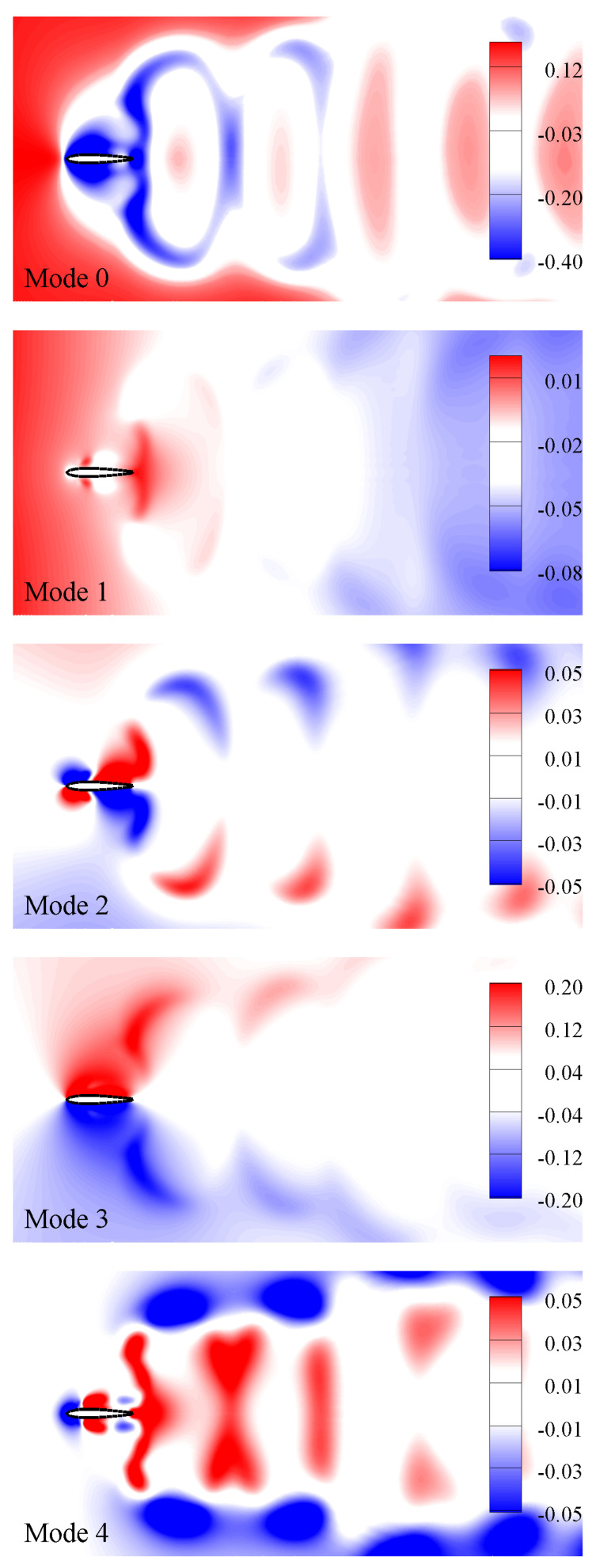
POD modes of pressure field at ${St}_{A}=0.33$.

Figure 7 presents the contributions of the modes towards the energy of this nonlinear dynamical system. It also indicates that the first four modes carry 98% of the total energy. Unlike the circular cylinder [70] and static airfoils at different angles of attack [32], where pressure modes are either pairwise symmetric or antisymmetric, the modes in the case of flapping airfoil corresponding to the power generation regime do not exhibit pairwise symmetry or antisymmetry. It is observed that after a symmetric mode about the center line (y=0), there is a pair of antisymmetric modes. In Figure 8, we show the mean and first four pressure POD modes. The mean mode (Mode 0) is symmetric as it shows a symmetric wake, causing zero mean lift, i.e., ${\bar{C}}_{L}=0$). Here, we compute these POD modes using 200 snapshots per oscillating cycle in order to include small-scale coherent structures of pressure field in the computational process.

### 5.2. Models of Hydrodynamic Forces and Moment

We develop the low-dimension models for fluidic forces (lift and drag) and moment (pitching moment) by projecting their respective full-dimensional models (given in Equation (9a)–(9c)) into space generated by pressure POD modes. It means that the modal forms of normal and axial, and pitching moment can be obtained by replacing the term *p* with its mode in Equation (13) as follows:

(18a) $$\begin{array}{c} F_{N}^{m}=-\oint_{c}\left({\bar{p}}^{s}\sin\left(\theta \right)+\sum_{j=1}^{M}a_{j}\left(t\right)\varphi_{j}^{s}\sin\left(\theta \right)\right)ds\\ =L_{o}^{0}+\sum_{j=1}^{M}a_{j}\left(t\right)L_{j}^{0} \end{array}$$

(18b) $$\begin{array}{c} F_{A}^{m}=-\oint_{c}\left({\bar{p}}^{s}\cos\left(\theta \right)+\sum_{j=1}^{M}a_{j}\left(t\right)\varphi_{j}^{s}\cos\left(\theta \right)\right)ds\\ =D_{o}^{0}+\sum_{j=1}^{M}a_{j}\left(t\right)D_{j}^{0} \end{array}$$

(18c) $$\begin{array}{c} M^{m}=\left(D_{o}^{1}-L_{o}^{1}\right)+\sum_{j=1}^{M}a_{j}\left(t\right)\left(D_{j}^{1}-L_{j}^{1}\right) \end{array}$$

where

(19a) $$L_{o}^{n}=-\oint_{c}{\left(x^{b}-x_{p}\right)}^{n}{\bar{p}}^{s}\sin\left(\theta \right)ds,$$

(19b) $$L_{j}^{n}=-\oint_{c}{\left(x^{b}-x_{p}\right)}^{n}\varphi_{j}^{s}\sin\left(\theta \right)ds,$$

(19c) $$D_{o}^{n}=-\oint_{c}{\left(y^{b}-y_{p}\right)}^{n}{\bar{p}}^{s}\cos\left(\theta \right)ds,$$

(19d) $$D_{j}^{n}=-\oint_{c}{\left(y^{b}-y_{p}\right)}^{n}\varphi_{j}^{s}\cos\left(\theta \right)ds,$$

where the subscripts *o* and *j* represent the decomposition of ${\bar{p}}^{s}$ and $\varphi_{j}^{s}$ into their sine and cosine components, respectively. The terms ${\bar{p}}^{s}$ and $\varphi_{j}^{s}$ represent the data on the surface of the foil surface from the $\bar{p}$ and $\varphi_{j}$, respectively. Besides, $x^{b}$ and $y^{b}$ are the coordinates of the nodal points on the body surface, which are, in general, functions of arc-length and time in the case of flapping foil, i.e., $x^{b}=x^{b}\left(s,t\right)$ and $y^{b}=y^{b}\left(s,t\right)$. The exponent ’*n*’ is an integer that is either 0 or 1. It is important to note that in the case of stationary bodies, terms $L_{j}^{0}$ and $D_{j}^{0}$ are called lift decomposition coefficient and drag decomposition coefficient, respectively, and make respective contributions to the lift and drag coefficients as the weight of the temporal coefficient [32,70].

Although the pressure temporal coefficient $a_{j}$ is predicted from the trained ANN model. However, in the training phase, we need $a_{j}$ *apriori*. To fulfill this requirement, we utilize orthogonality condition of the modes and derive the following relation in order to compute $a_{j}$ corresponding to each centralized snapshot: $a_{j}={\left(p^{\prime }\left(x,t_{j}\right),\varphi_{j}\left(x\right)\right)}_{H}$. We use data from ten oscillating cycles of the periodic steady-state phase. Two hundred snapshots per cycle are recorded that makes a total of 2000 snapshots in ten cycles. Then, we utilize these temporal coefficients for training the ANN model, as explained in the next subsection.

### 5.3. Long-Short-Term Neural Network

In 1997, Sepp Hochreiter and Jurgen Schmidhuber proposed the use of LSTM networks as a deep-learning approach for time series data [71]. LSTM networks are a type of recurrent neural network (RNN) that are designed to address the problem of vanishing gradients [72]. A recurrent neural network can process sequential data, where the output of a previous step is used as an input for the next step. However, traditional RNNs suffer from the problem of the vanishing gradients, where a gradient used to update the weights becomes very small as it propagates through the layers, making it difficult for the network to learn. The basic purpose of using LSTM networks is to overcome this critical problem. They consist of an LSTM cell, which includes three gates: An input gate, an output gate, and a forget gate. These gates control the flow of information in the LSTM cell and help prevent from the vanishing gradient problem. The input gate controls the flow of new information into the cell, the output gate controls the flow of information out of the cell, and the forget gate controls what information should be forgotten from the cell. These gates and the states within the LSTM cell can be calculated using the following equations:

(20a) $$\mathrm{Input}\;\mathrm{gate}:\;i_{t}=\sigma \left(W_{i}\cdot \left[y_{t-1},a_{t}\right]+b_{i}\right),$$

(20b) $$\tilde{C_{t}}=\tanh\left(W_{C}\cdot \left[y_{t-1},a_{t}\right]+b_{C}\right),$$

(20c) $$\mathrm{Forget}\;\mathrm{gate}:\;f_{t}=\sigma \left(W_{f}\cdot \left[y_{t-1},a_{t}\right]+b_{f}\right),$$

(20d) $$C_{t}=f_{t}*C_{t-1}+i_{t}*\tilde{C_{t}},$$

(20e) $$\mathrm{Output}\;\mathrm{gate}:\;o_{t}=\sigma \left(W_{o}\cdot \left[y_{t-1},a_{t}\right]+b_{o}\right),$$

(20f) $$y_{t}=o_{t}*\tanh\left(C_{t-1}\right).$$

The input of an LSTM cell is $\left[y_{t-1},a_{t}\right]$, where $y_{t-1}$ is the previous hidden state, and $a_{t}$ is the current input. Each gate is calculated by applying a sigmoid function to the dot product of the input $\left[y_{t-1},a_{t}\right]$ and the corresponding weight matrix plus bias. The sigmoid function outputs a value between 0 and 1.

The cell state $C_{t}$ is calculated by multiplying the previous cell state with the forget gate value and adding the input gate value multiplied by the candidate cell state computed by applying a tanh function to the dot product of the input $\left[y_{t-1},a_{t}\right]$ and the weight matrix $W_{c}$ plus bias $b_{c}$. The tanh function produces a value between −1 and 1 as the output. Finally, the hidden state $y_{t}$ is quantified by applying the output gate value to the tanh of the cell. TensorFlow, a python library for numerical computing [73], is utilized to train the neural network. The Adam optimizer; a method for updating the weights of the network, is employed during the training process. The Adam optimizer incorporates an exponential decay and learning rate adjustment in its governing mathematical equation, as described by [74].

**Figure 9:**
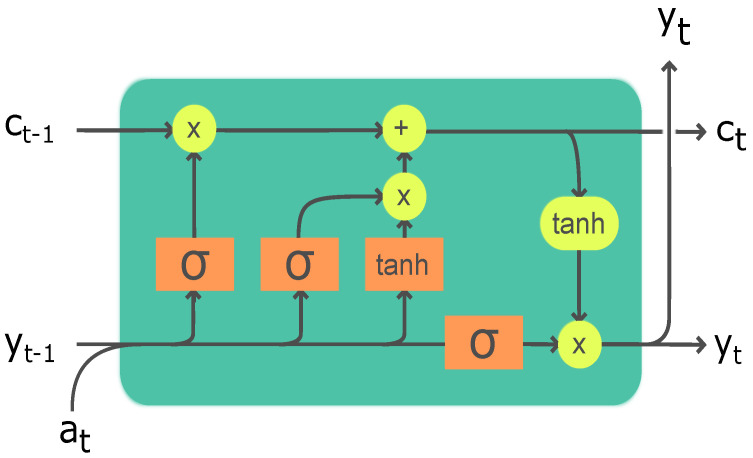
LSTM architecture with different gates. *a* represents the temporal coefficients where *y* represents the hidden state.

The input layer of the LSTM consists of the temporal coefficients at time level ‘*t*’. The input data $\left\{a_{1}^{\left(t\right)},a_{2}^{\left(t\right)},...,a_{n}^{\left(t\right)}\right\}\in R^{n}$ is then routed via hidden layers followed by the tanh activation function. The output layer consists of the temporal coefficients at time level ‘t+1’ $\left\{a_{1}^{\left(t+1\right)},a_{2}^{\left(t+1\right)},...,a_{n}^{\left(t+1\right)}\right\}\in R^{n}$. A schematic diagram to illustrate the training architecture is provided in Figure 9.

The data set is first divided into training and testing categories, with the training category making up 50% of the entire data set. The LSTM algorithm is then used to predict the next value in an initial sequence of data. The prediction process begins by providing the LSTM with the initial sequence of a length *t* and then, using the LSTM algorithm to predict the value at t+1. This prediction process is then repeated recursively, using the newly predicted value as an input for the next prediction. This process allows the LSTM to make predictions for future values in the sequence based on the patterns it learns from the training data.

**Figure 10:**
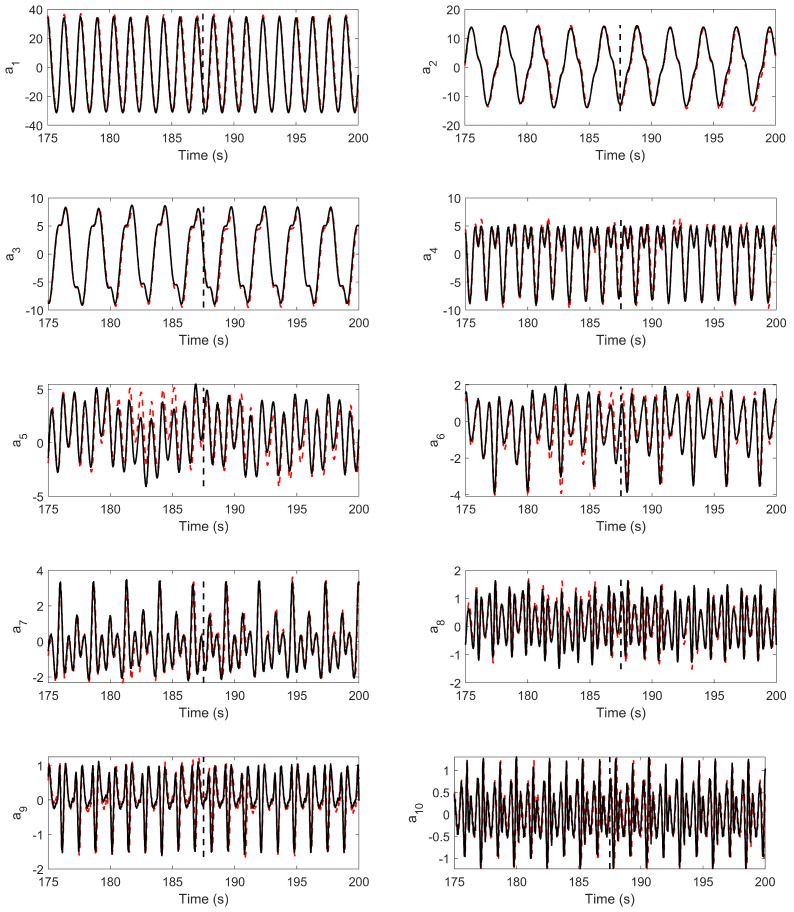
POD temporal coefficients: predicted (dashed line) and true (solid line). The vertical line splits the training and testing data.

**Figure 11:**
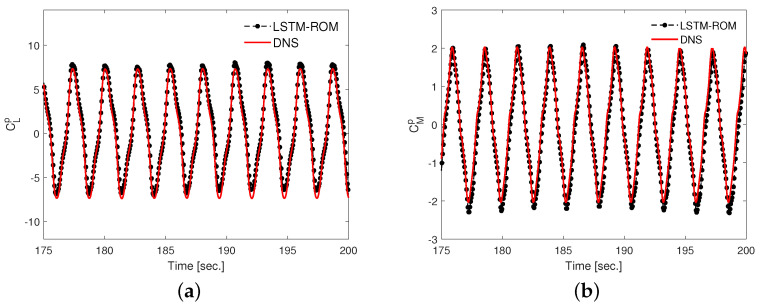
Reconstruction of pressure-based (**a**) lift coefficient $C_{L}^{p}$ and (**b**) moment coefficient $C_{M}^{p}$ using ten modes: predicted and true.

In our present work, we employ 200 hidden units with four hidden layers with 2000 epoch. In addition, dropout is used to prevent overfitting. After training, the network is utilized as a function to predict future time steps using the initial condition very similar to a conventional ROM, where the set of initial-valued ordinary differential equations is integrated over time. Figure 10 presents time histories of the predicted temporal coefficients along with their true values. The dashed vertical line serves as a boundary to separate the training data and testing data. The data used for testing is not meant for training and is hidden from the trained network. The trained LSTM model efficiently approximates the pressure POD coefficients, as evident from the comparison in Figure 10. We use the predicted temporal coefficients in the low-dimensional models of hydrodynamic forces and moment (Equation (18a), Equation (18b) and Equation (18c)) and reconstructed the pressure-based lift coefficient ($C_{L}^{\left\{m,p\right\}}$) and pitching moment coefficient ($C_{M}^{\left\{m,p\right\}}$) using ten POD modes. The comparison of time histories of $C_{L}^{\left\{m,p\right\}}$ and $C_{M}^{\left\{m,p\right\}}$ along with the data of direct numerical simulation (DNS) are presented in Figure 11. Moreover, we measure the accuracy of our model by computing the root-mean-square deviation (RMSD) between the predicted quantity and true DNS-based quantity. We notice that the RMSD values, in 10 oscillation cycles, containing both training and tested data (five cycles of each), are 13.76% and 8.26% in the lift and pitching moment coefficients, respectively. Thus, it exhibits 86.24% and 91.74% accuracy of the model for lift and pitching moment coefficients, respectively.

It is important to note that the pressure temporal coefficients are not only required for the reconstruction of hydrodynamic forces but also they are very useful for the development of efficient ROMs for shear flows. Noack et al. [30] reported that POD-based ROM (or POD-ROM) of incompressible shear flows with the inclusion of pressure term improves the accuracy of POD-ROMs. They emphasized that the lack of pressure term resulted in an amplitude error that could not be compensated by simply increasing the number of modes. Besides, Tallet et al. [75] further explained the importance of pressure terms in the development of an efficient POD-ROM for shear flows. They utilized the projection of momentum equations to compute both the velocity and pressure temporal coefficients. They examined the effectiveness of their methodology for POD-ROMs for a periodic flow past a circular cylinder and reconstructed the hydrodynamic coefficients as well as the Strouhal number, which provides a good agreement with those of the full-order model. Usually, projection of the pressure-Poisson equation is carried out, which results in a coupled velocity-pressure temporal coefficients system, and thus, it does not remain straightforward to be solved. This approach also stays limited to low-Reynolds number flows [21]. Moreover, [70] used the quadratic stochastic estimator to construct a relation between the temporal coefficients of pressure to the temporal coefficients of the velocity field through a mapping function. It helped us develop a ROM of the pressure field for flows over a cylinder at Re=100 instead of using the conventional approach [21]. Thus, estimating the temporal coefficients (of either velocity or pressure, or both) using deep-learning neural networks provides an effective solution to handle serious deficiencies of conventional techniques for developing efficient, accurate, and robust ROMs. Furthermore, the proposed LSTM model is not limited to pressure coefficients only, velocity coefficients can also be appended in training. Although this addition increases the amount of the data set and hence, the training time along with the tuning of training parameters.

## 6. Conclusions

In this study, a two-dimensional incompressible fluid flow around a NACA-0012 flapping foil is simulated and validated. The objective of this study is to develop a ROM for a power generating flapping foil using a deep neural networks model that works similarly to traditional ROMs. In the conventional ROMs of flow fields, the dynamical system along with the initial set of data, or precise initial conditions, are integrated over time for the prediction of future data, which may lead to erroneous results when prediction is required for a long-time duration. Here, we employ an LSTM network along with pressure POD to develop an efficient and a robust ROM for the pressure field over the surface of a flapping foil. The LSTM model is trained by temporal coefficients of a power generation system at St=0.33 and $A_{\alpha }=67^{\circ }$, which correspond to almost 40% power generation efficiency of a flapping foil. It is also tested with the data that are not used in the training process. Furthermore, we gauge the robustness of the new LSTM-based model to predict the future temporal coefficients for a long time duration that is far beyond the training time interval. The results show a good efficiency and robustness of the proposed LSTM model, which can accurately predict the temporal coefficients and corresponding hydrodynamic forces and moment. It also demonstrates a good agreement with the true values obtained from the full-order simulation. We find 86.24% and 91.74% accuracy of the model in the reconstruction of lift and pitching moment coefficients, respectively. Therefore, the LSTM model can be a viable tool for the prediction of a flow field for a long-time duration. It is also more efficient and accurate compared to the conventional Galerkin projection-based POD-ROM techniques.

## Data Availability

Data can be provided by the authors upon reasonable requests.
